# Oxidative Stress and Inflammatory Responses in Horses Naturally Infected with *Theileria equi*

**DOI:** 10.3390/ph19081208

**Published:** 2026-08-01

**Authors:** Vito Biondi, Pietro Gambadauro, Fabio Bruno, Valentina Palmieri, Patrizia Licata, Diego Antonio Sicuso, Annamaria Passantino, Michela Pugliese

**Affiliations:** Department of Veterinary Sciences, University of Messina—Via Giovanni Palatucci, 98168 Messina, Italy; vbiondi@unime.it (V.B.); pietro.gambadauro@studenti.unime.it (P.G.); valentina.palmieri1@studenti.unime.it (V.P.); patrizia.licata@unime.it (P.L.); diego.sicuso@studenti.unime.it (D.A.S.); annamaria.passantino@unime.it (A.P.); michela.pugliese@unime.it (M.P.)

**Keywords:** *Theileria equi*, equine piroplasmosis, oxidative stress, malondialdehyde, glutathione, IFN-γ, TNF-α, inflammation, antioxidant status, horse

## Abstract

Equine piroplasmosis, caused by the intraerythrocytic protozoan *Theileria equi*, is a globally distributed tick-borne disease characterized by persistent infection and variable clinical manifestations. Although oxidative stress has been implicated in the pathogenesis of several hemoprotozoan diseases, information regarding redox imbalance and its relationship with inflammatory responses in horses naturally infected with *T. equi* remains limited. This study aimed to evaluate oxidative stress, antioxidant status, inflammatory cytokines, and clinicopathological alterations in horses naturally infected with *T. equi*. Thirty-five horses naturally infected with *T. equi* and twenty clinically healthy horses were enrolled. Infection was confirmed by clinical evaluation, hematological and biochemical analyses, an indirect fluorescent antibody test, and polymerase chain reaction. Serum concentrations of interferon-gamma, tumor necrosis factor-alpha, reduced glutathione, and malondialdehyde were assayed. Infected horses showed significantly lower platelet counts and significantly higher serum activities of aspartate aminotransferase than controls. Serum interferon-gamma and tumor necrosis factor concentrations were significantly increased, indicating persistent inflammatory activation. Infected horses also exhibited significantly higher malondialdehyde concentrations and lower reduced glutathione levels, consistent with enhanced lipid peroxidation and impaired antioxidant defenses. Correlation analyses revealed a negative association between reduced glutathione and white blood cell count and between malondialdehyde and red blood cell count, whereas malondialdehyde concentrations were positively associated with clinical score. Chronic *T. equi* infection is associated with oxidative stress, depletion of antioxidant defenses, and sustained inflammatory responses. The interaction between oxidative stress and inflammation may contribute to hematological abnormalities and disease expression.

## 1. Introduction

Theileriosis is a tick-borne disease of equids caused by the intraerythrocytic apicomplexan parasite *Theileria equi*. Transmission occurs primarily through ixodid ticks belonging to the genera *Dermacentor*, *Hyalomma*, and *Rhipicephalus*, although vertical and iatrogenic transmission have also been documented. Following infection, *T. equi* initially invades leukocytes before infecting erythrocytes, a biological feature that contributes to its persistence within the host and distinguishes it from other equine hemoparasites [1].

*T. equi* is the most prevalent causative agent of equine piroplasmosis worldwide. Approximately 90% of the global equine population is estimated to reside in endemic regions, and a recent systematic review reported a pooled prevalence of 29.4% [2,3]. This widespread distribution is largely attributable to the parasite’s ability to establish persistent infections, allowing infected animals to serve as long-term reservoirs of transmission. In addition, reduced susceptibility to currently available therapeutic agents has been reported in some isolates, underscoring the need for a deeper understanding of disease pathogenesis and host–parasite interactions [3].

Clinical manifestations typically develop within 12–19 days after infection and range from severe acute disease, characterized by fever, hemolytic anemia, jaundice, and pigmenturia, to chronic or subclinical infections associated with poor performance, weight loss, and intermittent clinical abnormalities [4,5,6]. Although mortality is primarily associated with acute disease, chronically infected horses constitute an important reservoir for parasite maintenance and dissemination. Consequently, the substantial economic and sanitary impact of EP has prompted the implementation of strict international regulations that require testing of equids before movement and export [6,7].

Oxidative stress has emerged as a key pathogenic mechanism in a variety of infectious and inflammatory disorders. Excessive production of reactive oxygen species (ROS) can induce lipid peroxidation, protein oxidation, DNA damage, and cellular dysfunction, ultimately contributing to tissue injury and disease progression [8]. In hemoprotozoan infections, oxidative imbalance may arise from both parasite metabolism and the host inflammatory response, thereby exacerbating erythrocyte destruction and tissue damage.

Studies in bovine and canine babesiosis have consistently demonstrated increased oxidative stress and impaired antioxidant defenses during infection [9,10,11]. In contrast, information regarding oxidative status in horses naturally infected with *T. equi* remains limited, and only a small number of oxidative biomarkers have been investigated to date [12,13]. A better understanding of redox alterations in equine piroplasmosis could improve current knowledge of disease pathophysiology and facilitate the identification of novel prognostic biomarkers and therapeutic targets.

Therefore, the present study aimed to evaluate oxidative stress and antioxidant status in horses naturally infected with *T. equi* by assessing selected biomarkers of oxidative damage and antioxidant defense. Additionally, the relationship between oxidative imbalance and clinicopathological alterations was investigated to further elucidate the contribution of oxidative stress to the pathophysiology of equine piroplasmosis.

## 2. Results

The infected horses (TEQ) had a mean age of 11.83 ± 7.42 years. All horses were seropositive based on the indirect fluorescent antibody (IFA) test, with antibody titers ranging from 1:50 to 1:1600. In addition, 6/35 (17.1%) horses tested positive on blood smear examination, whereas 29/35 (82.9%) were PCR-positive. All horses in the TEQ group exhibited mild, non-specific clinical signs, including poor performance (31.4%; 11/35), mild weight loss (28.6%; 10/35), lethargy (42.9%; 15/35), anorexia (22.9%; 8/35), and pale mucous membranes (51.4%; 18/35). The mean value of the clinical scoring system (CSS) employed to classify the clinical findings was 2.30 ± 1.40.

Comparison of hematological variables between the two groups revealed a significant difference in PLT after Benjamini–Hochberg correction for multiple testing (BH-adjusted *p* = 0.011). In contrast, the differences observed in RBC, Hgb, and PCV were no longer statistically significant after adjustment (Table 1). Comparison of biochemical variables between the two groups showed that AST remained significantly higher in the TEQ group after Benjamini–Hochberg correction for multiple testing (BH-adjusted *p* = 0.016), whereas the increase in ALT was no longer statistically significant following adjustment (Table 1).

**Figure 1:**
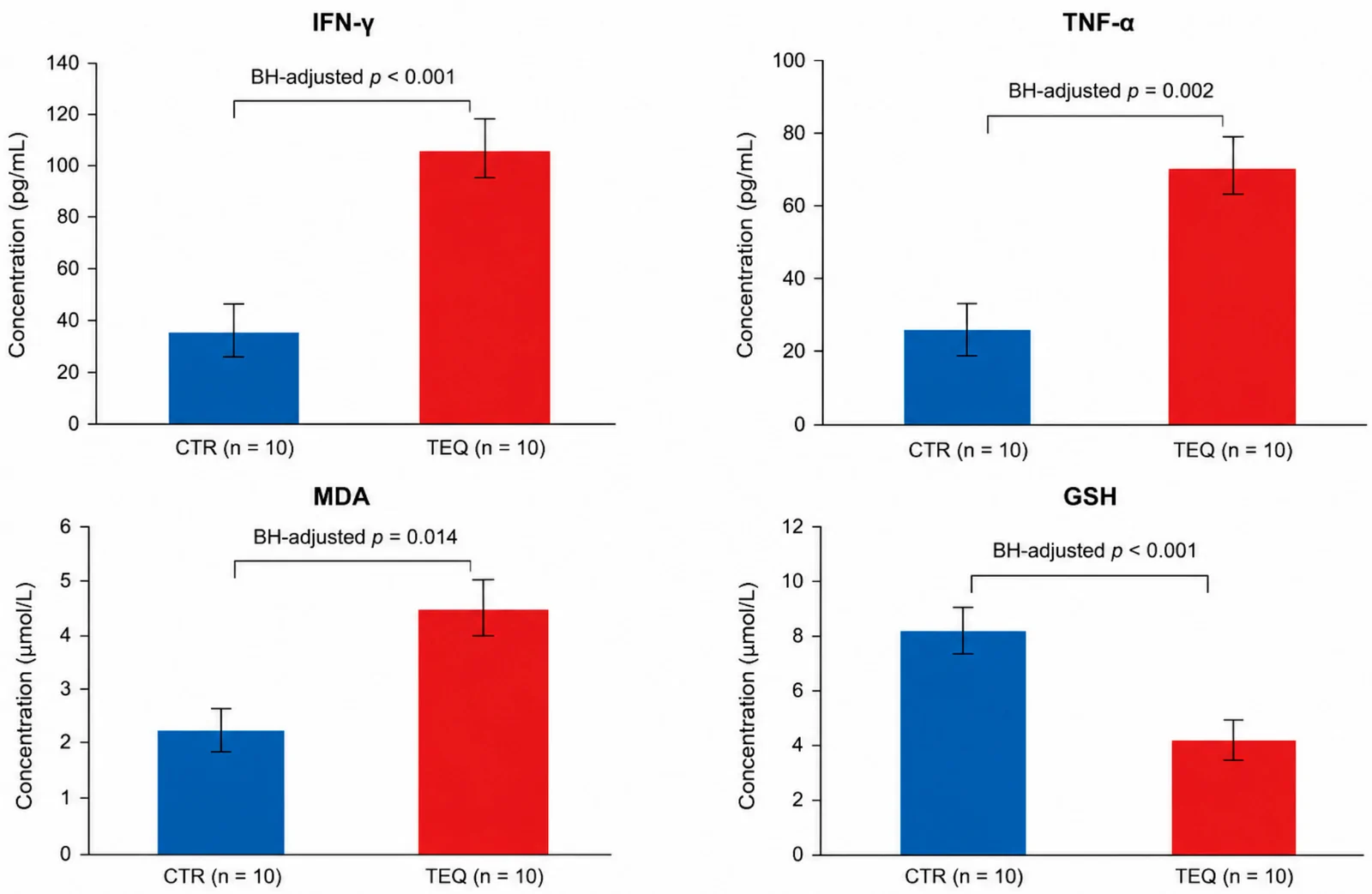
Comparison of serum concentrations of IFN-γ, TNF-α, MDA, and GSH between the control (CTR) and TEQ groups. Horses in the TEQ group exhibited significantly higher concentrations of IFN-γ, TNF-α, and MDA, whereas GSH concentrations were significantly lower compared with the CTR group. Statistical significance was assessed using the Benjamini–Hochberg correction for multiple testing (BH-adjusted *p* < 0.001 for IFN-γ and GSH, BH-adjusted *p* = 0.002 for TNF-α, and BH-adjusted *p* = 0.014 for MDA). Data are presented as mean ± SD. TEQ, horses infected by *T. equi*; CTR, healthy horses; IFN-γ, interferon-gamma; TNF-α, tumor necrosis factor; GSH, reduced glutathione; MDA, malondialdehyde.

Horses in the TEQ group exhibited significantly higher concentrations of IFN-γ, TNF-α, and MDA, and significantly lower GSH concentrations than horses in the CTR group. These differences remained statistically significant after Benjamini–Hochberg correction for multiple testing (BH-adjusted *p* < 0.001 for IFN-γ and GSH, BH-adjusted *p* = 0.002 for TNF-α, and BH-adjusted *p* = 0.014 for MDA) (Table 2; Figure 1).

The clinical scoring system (CSS) employed to classify the clinical findings was significantly higher in the TEQ group than in the CTR group (2.30 ± 1.40 vs. 0 ± 0), and the difference remained significant after Benjamini–Hochberg correction for multiple testing (BH-adjusted *p* < 0.001).

Spearman’s correlation analyses of TEQ showed a negative correlation between GSH and the number of WBC (*p* = 0.04; r = −0.86). Also, a negative correlation was detected between MDA and RBC (*p* = 0.021; r = −0.55). MDA was positively correlated with clinical score (*p* = 0.04; r = 0.44) (Table 3).

## 3. Discussion

Horses included in the TEQ group exhibited only mild and non-specific clinical signs, including lethargy, poor performance, weight loss, anorexia, and pale mucous membranes. The relatively low clinical score supports the presence of a chronic or carrier state of *T. equi* infection, which is consistent with the epidemiological behavior of the parasite and its remarkable ability to establish lifelong persistent infections in equids [5]. In endemic areas, carrier horses represent the most important reservoir of infection, maintaining parasite circulation within both equine populations and tick vectors. Consequently, even animals showing minimal clinical alterations may contribute significantly to disease transmission.

Despite the mild clinical presentation, infected horses exhibited hematological alterations, with thrombocytopenia remaining significant after Benjamini–Hochberg correction for multiple testing. Although lower RBCs, hemoglobin concentration, and packed cell volume were observed in the TEQ group, these differences did not remain statistically significant after adjustment for multiple comparisons. Nevertheless, this trend is consistent with previous reports describing anemia and thrombocytopenia as common hematological abnormalities in equine piroplasmosis [5,13]. The reduction in erythrocyte parameters is likely multifactorial. In addition to the direct destruction of infected erythrocytes during parasite replication, immune-mediated mechanisms, including erythrophagocytosis by activated macrophages and splenic sequestration, have been proposed as major contributors to anemia development. Furthermore, oxidative injury to erythrocyte membranes may increase cell fragility and accelerate erythrocyte clearance from circulation.

Similarly, thrombocytopenia is frequently reported in *T. equi*-infected horses and may result from increased platelet consumption, splenic pooling, immune-mediated destruction, or disseminated endothelial activation [5,14]. Although the exact mechanisms remain incompletely understood, platelet alterations have been associated with inflammatory and vascular changes occurring during hemoprotozoan infections. Interestingly, thrombocytopenia was evident even in horses with only mild clinical signs, suggesting that hematological disturbances may persist during chronic infection and could serve as sensitive indicators of disease activity.

The significantly higher serum AST activity observed in infected horses, which remained significant after Benjamini–Hochberg correction for multiple testing, suggests the presence of tissue injury. Although ALT activity was also higher in the TEQ group, this difference did not remain statistically significant after adjustment for multiple comparisons. Increased AST activity has previously been reported in equine piroplasmosis and may reflect hepatic involvement, muscular damage, or both [6]. Several pathogenic mechanisms may contribute to these alterations, including hypoxic injury secondary to anemia, inflammatory cytokine-mediated tissue damage, and oxidative stress-induced cellular dysfunction. Although the observed biochemical changes were moderate, they support the presence of subclinical tissue involvement during chronic *T. equi* infection.

One of the most relevant findings of the present study was the marked increase in IFN-γ and TNF-α concentrations in infected horses. These cytokines are key mediators of the Th1 immune response and play a crucial role in host defense against intracellular protozoan pathogens [15]. Interferon-gamma is primarily produced by activated T lymphocytes and natural killer cells and promotes macrophage activation, antigen presentation, and intracellular parasite killing [16]. Previous studies have demonstrated increased expression of IFN-γ in horses naturally infected with *T. equi*, with cytokine levels correlating positively with parasitemia and disease severity [17]. Although previous studies have reported positive associations between IFN-γ concentrations, parasitemia, and disease severity, the low and heterogeneous parasite burden observed in the present cohort precluded a similar analysis. Therefore, the elevated IFN-γ concentrations observed in the present study may reflect the activation of cell-mediated immunity involved in limiting parasite persistence.

The simultaneous increase in IFN-γ and TNF-α strongly suggests the persistence of a chronic inflammatory state in infected horses. Similar cytokine patterns have been reported in several protozoan infections, where long-term antigenic stimulation maintains continuous activation of the immune system. While this response may contribute to parasite control, it may also promote the generation of reactive oxygen species (ROS), linking inflammation with oxidative stress and tissue damage.

Indeed, the oxidative stress profile observed in the present study provides compelling evidence of redox imbalance in horses infected with *T. equi*. Significantly increased MDA concentrations, accompanied by reduced GSH levels, indicate enhanced lipid peroxidation and depletion of antioxidant defences. Oxidative stress is increasingly recognized as a major component of the pathogenesis of parasitic diseases, resulting from the imbalance between ROS production and antioxidant capacity [18,19,20]. During infection, ROS are generated both by activated immune cells and by parasite-induced metabolic alterations. Although these reactive molecules are essential for pathogen elimination, excessive ROS production can damage host tissues and contribute to disease progression.

The increase in MDA observed in the infected horses is particularly noteworthy because MDA is one of the most widely used biomarkers of lipid peroxidation. Elevated MDA concentrations indicate oxidative damage to cellular membranes and have previously been reported in several infectious and inflammatory diseases, including hemoprotozoan infections [6,21]. The erythrocyte membrane is especially susceptible to oxidative damage because of its high content of polyunsaturated fatty acids and continuous exposure to oxygen. Consequently, increased lipid peroxidation may compromise membrane integrity, increase osmotic fragility, and ultimately promote hemolysis.

Conversely, GSH represents one of the most important intracellular antioxidant molecules and constitutes a primary defence mechanism against ROS-mediated injury. Reduced glutathione protects proteins, lipids, and nucleic acids from oxidative damage by directly scavenging free radicals and serving as a substrate for antioxidant enzymes. During parasitic infections, increased ROS production results in accelerated GSH consumption and conversion to its oxidized form (GSSG) [22]. Therefore, the significantly lower GSH concentrations observed in infected horses suggest that antioxidant reserves were insufficient to counterbalance oxidative challenges generated during chronic infection.

The correlation analyses further support the biological relationship between inflammation, oxidative stress, and hematological alterations. The strong negative correlation detected between GSH and WBC suggests that activation of leukocytes contributes substantially to antioxidant depletion. Activated granulocytes and macrophages generate large amounts of ROS during the respiratory burst as part of the innate immune response [19,23]. Consequently, increased leukocyte activity may accelerate glutathione consumption and exacerbate oxidative imbalance.

Furthermore, the negative correlation between MDA and RBC suggests a direct association between lipid peroxidation and erythrocyte loss. This finding supports the hypothesis that oxidative damage contributes to erythrocyte membrane instability and premature destruction. Similar mechanisms have been described in bovine and canine babesiosis, where oxidative injury is considered a major factor involved in anemia development [22,24]. These findings suggest that oxidative stress may represent an additional mechanism involved in the hematological alterations associated with equine theileriosis. Interestingly, MDA concentrations were positively correlated with clinical score, indicating that oxidative damage increased with the severity of clinical manifestations. Although the horses included in this study displayed only mild signs, this association suggests that oxidative stress may influence disease expression and progression. Similar observations have been reported in other parasitic diseases, where higher levels of oxidative biomarkers are associated with poorer clinical outcomes and more severe inflammatory responses [18].

Overall, the present findings suggest that chronic *T. equi* infection is associated with a complex interplay between persistent inflammation and oxidative stress. Increased production of pro-inflammatory cytokines, depletion of antioxidant defenses, and enhanced lipid peroxidation appear to be closely interconnected mechanisms contributing to tissue injury, erythrocyte destruction, and clinical manifestations. These results support previous evidence suggesting that oxidative stress plays a central role in the pathophysiology of EP [25] and indicate that biomarkers such as MDA and GSH may provide useful information regarding disease activity, progression, and response to treatment. Further studies involving larger populations and different clinical stages of infection are warranted to clarify the prognostic significance of these biomarkers and to evaluate whether antioxidant-based therapeutic strategies may complement conventional antiparasitic treatments.

An additional aspect worthy of consideration is the potential interplay between oxidative stress and parasite persistence. *T. equi* possesses sophisticated immune-evasion mechanisms that allow the parasite to survive within the host for prolonged periods despite an active immune response. Chronic exposure to parasite antigens may induce sustained activation of inflammatory pathways, resulting in continuous ROS production and progressive exhaustion of antioxidant reserves. This persistent oxidative environment may contribute to host tissue injury and potentially influence the host–parasite equilibrium, thereby favoring long-term parasite persistence. Similar mechanisms have been described in other chronic protozoal diseases, where oxidative stress participates in both pathogen control and disease pathogenesis [26].

Moreover, the observed reduction in GSH concentrations suggests that antioxidant defenses become compromised even in horses exhibiting relatively mild clinical signs. This finding may have important clinical implications because oxidative imbalance could precede the development of overt hematological or biochemical abnormalities. Consequently, oxidative stress biomarkers may provide additional information beyond conventional laboratory parameters, potentially enabling earlier detection of disease activity and subclinical progression. The assessment of biomarkers such as MDA and GSH could therefore complement routine diagnostic approaches and contribute to a more comprehensive evaluation of infected animals.

The potential role of antioxidant supplementation as an adjunctive therapeutic approach warrants further investigation. Antioxidants, including vitamin E, selenium, N-acetylcysteine, and other redox-modulating compounds, have shown beneficial effects in several infectious and inflammatory conditions by reducing oxidative damage and supporting cellular defense mechanisms [26,27]. Although antiparasitic treatment remains the cornerstone of equine piroplasmosis management, modulation of oxidative stress may help attenuate tissue injury, improve clinical recovery, and preserve erythrocyte integrity. Nevertheless, controlled clinical studies are required to determine whether antioxidant interventions can effectively improve outcomes in horses naturally infected with *T. equi* [15].

Despite the promising findings, several limitations should be considered when interpreting the results of the present study. First, because this investigation was based on naturally occurring infections, the duration of infection could not be reliably determined. Moreover, although parasitemia was assessed microscopically in all infected horses, parasite burden was generally low and heterogeneous among individuals, and no quantitative molecular assay was performed. Consequently, it was not possible to investigate the relationship between parasite burden and oxidative stress biomarkers. Likewise, although all infected horses were seropositive by indirect fluorescent antibody test (IFAT), antibody titers reflected exposure rather than parasite burden or disease severity and therefore could not be used to stratify animals according to infection intensity.

Furthermore, the assessment of oxidative status was limited to malondialdehyde (MDA) and reduced glutathione (GSH). Although these are well-established indicators of lipid peroxidation and antioxidant capacity, they provide only a partial characterization of redox homeostasis. The inclusion of additional biomarkers, such as superoxide dismutase (SOD), catalase (CAT), glutathione peroxidase (GPx), total antioxidant status (TAS), total oxidant status (TOS), and the oxidative stress index (OSI), would allow a more comprehensive evaluation of oxidative balance during *Theileria equi* infection.

Another limitation is the relatively small sample size and the inclusion of horses predominantly affected by chronic infection with mild clinical manifestations, which may limit the generalizability of the findings to animals with acute or severe equine piroplasmosis. In addition, the cross-sectional design precludes the assessment of temporal changes in oxidative stress biomarkers and does not allow causal relationships among parasite infection, inflammation, oxidative stress, and clinicopathological alterations to be established. Finally, despite the selection of horses maintained under comparable environmental and management conditions, individual factors such as age, nutritional status, and genetic background may have contributed to the observed variability in oxidative and inflammatory responses.

Future studies involving larger populations, different clinical stages of infection, longitudinal follow-up, and quantitative molecular techniques, such as real-time PCR, are warranted to better characterize the relationships among parasite burden, host immune responses, and oxidative stress. Such investigations may also help determine the prognostic value of redox biomarkers and clarify whether antioxidant therapies could represent a useful adjunct to conventional antiparasitic treatment in horses naturally infected with *T. equi*.

## 4. Materials and Methods

### 4.1. Animals and Procedures

The study was conducted using blood samples collected during routine sanitary inspections performed by the official veterinarian of the local district authority as part of standard animal welfare and official disease-monitoring programs, including mandatory surveillance for Equine Infectious Anemia and routine vaccination controls. Because blood collection was required exclusively for routine diagnostic and prophylactic purposes to assess the health status of the animals, no additional procedures were performed specifically for research. Consequently, ethical approval was not required, as all sampling procedures were non-invasive, formed part of routine veterinary practice, and were performed by qualified personnel in accordance with current Italian and European legislation on animal welfare. Nevertheless, horse owners were fully informed about the objectives of the study, and written informed consent for the secondary use of the samples was obtained before enrollment. The confidentiality of all personal data was strictly maintained throughout the study.

All enrolled horses originated from private farms located in Sicily (southern Italy) and were maintained under comparable environmental, husbandry, and management conditions.

This observational study was based on naturally occurring cases of *Theileria equi* infection; therefore, no *a priori* sample size calculation was performed. Instead, all horses meeting the predefined inclusion criteria during the study period were consecutively enrolled. Because the study relied on naturally infected animals, equal group sizes were not feasible.

To minimize potential confounding effects, all enrolled horses were screened at the time of enrolment by the indirect fluorescent antibody test (IFAT) for *Babesia caballi* and *Anaplasma phagocytophilum*, and animals testing positive for either pathogen were excluded from the study.

The inclusion criteria for the infected group were: (i) compatible clinical findings; (ii) positive results for both IFAT and polymerase chain reaction (PCR) for *T. equi*; (iii) no antiparasitic or supportive treatment before sample collection; and (iv) availability of complete clinical and laboratory records. Horses with concurrent systemic diseases or incomplete clinical records were excluded. Control horses were required to be clinically healthy, PCR-negative for *T. equi*, and to have normal hematological and biochemical findings.

Affected horses had a documented history of tick exposure and exhibited clinical signs including lethargy, anorexia, hyperthermia, and dark yellow urine suggestive of bilirubinuria. None had received antiparasitic or supportive treatment before sample collection. Based on their clinical presentation, low clinical scores, and the absence of severe hemolytic crises or other manifestations typically associated with acute equine piroplasmosis, infected horses were classified as chronically infected animals with mild clinical disease rather than asymptomatic carriers. This distinction is clinically relevant because chronic *T. equi* infection may persist for prolonged periods while causing subtle clinical abnormalities and maintaining a reservoir for parasite transmission.

The diagnosis of *T. equi* infection was established based on compatible clinical findings together with positive IFAT and PCR results [28]. Peripheral blood smear examination was also performed to estimate parasitemia; however, because of its limited sensitivity in chronically infected horses, it was not considered an inclusion criterion [28].

The infected group (TEQ) comprised 35 horses fulfilling all inclusion criteria, whereas the control group (CTR) consisted of 20 clinically healthy horses selected from the same farms as the infected animals. The health status of control horses was confirmed by physical examination, hematological and biochemical analyses, peripheral blood smear evaluation, negative PCR results for *T. equi*, and negative IFAT results. Control horses were comparable to infected horses with respect to age, sex, breed, and management conditions, thereby minimizing the influence of environmental and husbandry-related confounding factors.

The characteristics of the study population and the criteria used for group classification are summarized in Table 4.

### 4.2. Clinical Scoring System (CSS)

A scoring system was employed to classify the clinical findings. Four clinical variables (poor performance, hyperthermia, leukocytosis, and mucous membrane status) were evaluated. Each variable was assigned a score of 0 (absence of the clinical sign or normal finding) or 1 (presence of the clinical sign or abnormal finding). The total CSS ranged from 0 to 4, with higher scores indicating greater clinical impairment (Table 5).

For hematological evaluation, blood samples were collected from the jugular vein into vacuum tubes (Terumo Co., Tokyo, Japan). Samples intended for hematological analysis and PCR testing were collected into tubes containing K_2_-EDTA as an anticoagulant, whereas additional blood samples were collected into additive-free tubes for serum separation and subsequent biochemical and oxidative stress analyses.

Peripheral blood smears were prepared from capillary blood obtained from the upper lip vein, air-dried, stained with Giemsa, and examined microscopically. Parasitemia was assessed by counting infected erythrocytes under oil immersion at ×1000 magnification [14]. Samples were considered negative when no parasites were detected in 50 microscopic fields.

Hematological analyses were performed using an automated multiparametric hematology analyzer (ProCyte Dx^®^, IDEXX Laboratories, Westbrook, ME, USA) on EDTA-anticoagulated blood samples. The parameters evaluated included red blood cell count (RBC), packed cell volume (PCV), hemoglobin concentration (Hb), mean corpuscular volume (MCV), mean corpuscular hemoglobin (MCH), mean corpuscular hemoglobin concentration (MCHC), red blood cell distribution width (RDW-SD and RDW-CV), white blood cell count (WBC), lymphocyte count, granulocyte count, mid-sized cell count (MID), platelet count (PLT), mean platelet volume (MPV), platelet distribution width (PDW), and plateletcrit (PCT).

Serum biochemical analyses included aspartate aminotransferase (AST), alanine aminotransferase (ALT), total protein (TP), albumin (Alb), blood urea nitrogen (BUN), and creatinine (CREA).

Genomic DNA was extracted from EDTA-anticoagulated blood samples using a commercial extraction kit according to the manufacturer’s instructions.

Serum samples were obtained by allowing whole blood to clot at room temperature for approximately 20 min, followed by centrifugation at 1000× *g* for 20 min. The obtained serum was collected and stored at −80 °C until analysis. Interferon-gamma (IFN-γ) concentrations were quantified using a commercially available sandwich ELISA kit (Horse IFNg ELISA Kit, AssayGenie, Dublin, Ireland) according to the manufacturer’s instructions and color development was achieved using tetramethylbenzidine (TMB) substrate. Tumor necrosis factor-alpha (TNF-α) concentrations were determined using a quantitative sandwich ELISA kit (Horse Tumor Necrosis Factor α ELISA Kit, Cusabio, Houston, TX, USA) according to the manufacturer’s guidelines. Reduced glutathione (GSH) levels were measured using a commercial ELISA kit (Horse Glutathione ELISA Kit, AssayGenie, Dublin, Ireland) according to the manufacturer’s protocols. Optical density was recorded at 450 nm (570 nm reference for TNF-α) using the BIORAD 680 microplate reader (BIORAD Laboratories, Segrate, Italy). Lipid peroxidation was assessed by measuring malondialdehyde (MDA) concentrations using the thiobarbituric acid reactive substances (TBARS) assay. Serum samples were mixed with phosphoric acid and thiobarbituric acid reagent and incubated at 90 °C for 1 h to allow formation of the MDA-TBA adduct. Following cooling on ice, aliquots of standards and samples were transferred to a 96-well microplate. Absorbance was measured at 535 and 572 nm using a microplate reader BIORAD 680 (BIORAD Laboratories, Italy), and MDA concentrations were determined from calibration curves prepared using hydrolyzed tetramethoxypropane standards. Results were expressed as TBARS equivalents and used as a bioindicator of oxidative stress [29,30,31].

### 4.3. Statistical Analysis

All statistical analyses were performed using SPSS for Windows (version 17.0; SPSS Inc., Chicago, IL, USA). Data are presented as mean ± standard deviation (SD). Data normality was assessed using the Kolmogorov–Smirnov test. Comparisons between the TEQ and CTR groups were conducted using Welch’s t-test for normally distributed variables and the Mann–Whitney U test for non-normally distributed variables. Associations between oxidative stress biomarkers and hematological or biochemical parameters were evaluated using Spearman’s rank correlation coefficient. To account for multiple comparisons, *p*-values from the between-group comparisons were adjusted using the Benjamini–Hochberg procedure to control the false discovery rate (FDR) at 5%. The Benjamini–Hochberg correction was applied separately to each family of related outcomes (hematological and biochemical variables, and oxidative stress biomarkers/cytokines). Correlation analyses were considered exploratory and are therefore reported using unadjusted *p*-values. A two-tailed *p*-value < 0.05 was considered statistically significant, and for multiple comparisons, statistical significance was based on Benjamini–Hochberg-adjusted *p*-values (FDR < 0.05).

## 5. Conclusions

The integration of inflammatory and oxidative biomarkers may provide a useful framework for monitoring disease evolution and treatment response. Longitudinal investigations evaluating changes in cytokine profiles, antioxidant status, and parasitemia over time would further clarify the dynamic relationship between host immunity, oxidative stress, and parasite persistence in equine piroplasmosis.

## Figures and Tables

**Table 1:** Hematological and biochemical variables in horses infected with *Teileria Equi* (TEQ) and in healthy horses (CTR).

Variables	Unit	TEQ	CTR	*p*-Value
		Mean ± SD	Mean ± SD	
RBC	(10^6^/µL)	6.60 ± 1.00	7.74 ± 2.96	Ns
Hgb	(g/dL)	10.70 ± 1.20	17.05 ± 3.60	Ns
PCV	(%)	40.88 ± 12.20	44.80 ± 4.38	Ns
WBC	(10^9^/µL)	8.78 ± 2.40	8.42 ± 2.34	Ns
PLT	(10^9^/µL)	107.00 ± 36.57	238.95 ± 150.71	0.011
ALT	(U/L)	149.50 ± 129.59	91.50 ± 63.53	Ns
AST	(U/L)	34.36 ± 13.94	23.38 ± 11.00	0.016
BUN	(mg/dL)	18.70 ± 2.54	16.35 ± 2.98	Ns
CREA	(mg/dL)	1.70 ± 0.21	1.40 ± 0.15	Ns
ALB	(g/dL)	3.80 ± 1.80	3.50 ± 1.60	Ns
TP	(g/dL)	7.10 ± 0.43	6.60 ± 0.25	Ns

TEQ, horses infected with *T. equi*; CTR, healthy horses; RBCs, red blood cells; Hgb, hemoglobin; PCV, packed-cell volume; WBCs, white blood cells; PLTs, platelets; BUN, blood urea nitrogen; CREA, creatinine; AST, aspartate aminotransferase; ALT, alanine aminotransferase; ALB, albumin; TP, total protein; Ns, not significant.

**Table 2:** Variables with normal distribution: IFN-γ, TNF-α, GSH and MDA concentrations in TEQ and CTR groups.

Variables	Unit	TEQ	CTR	*p*-Value
		Mean ± DS	Mean ± DS	
IFN-γ	pg/mL	20.01 ± 1.48	7.74 ± 2.96	<0.001
TNF-α	pg/mL	19.32 ± 1.16	17.00 ± 3.60	0.002
GSH	ng/mL	61.41 ± 2.08	78.90 ± 12.20	<0.001
MDA	µM	21.97 ± 6.40	12.12 ± 4.10	0.014

TEQ, horses infected by *T. equi*; CTR, healthy horses; IFN-γ, interferon-gamma; TNF-α, tumor necrosis factor; GSH, reduced glutathione; MDA, malondialdehyde.

**Table 3:** Correlation between oxidative stress and hematochemical variables in the TEQ group (Spearman rank order).

Variables	CS	RBC	Hgb	PCV	WBC	PLT	ALT	AST	BUN	Crea	ALB	TP
IFN-γ	0.17	−0.10	−0.10	−0.10	−0.17	0.12	−0.03	−0.01	0.09	−0.01	0.06	−0.06
TNF-α	0.04	0.03	−0.24	−0.24	0.04	0.05	−0.15	0.05	−0.03	0.05	0.30	0.35
GSH	0.48	0.02	0.27	0.27	−0.86 *	0.15	−0.21	0.05	0.14	0.05	0.06	−0.1
MDA	0.44 **	−0.55 *	0.20	0.03	0.15	−0.10	−0.30	0.30	−0.11	−0.04	0.27	0.20

IFN-γ, interferon-gamma; TNF-α, tumor necrosis factor; GSH, reduced glutathione; MDA, malondialdehyde; RBCs, red blood cells; Hgb, hemoglobin; PCV, packed-cell volume; WBCs, white blood cells; PLTs, platelets; BUN, blood urea nitrogen; CREA, creatinine; AST, aspartate aminotransferase; ALT, alanine aminotransferase; ALB, albumin; TP, total protein; * statistically significant; ** highly statistically significant.

**Table 4:** Classification criteria and baseline characteristics of infected horses with *Theileria equi* (TEQ) and control horse groups (CTR).

Criteria	TEQ (n = 35)	CTR (n = 20)
Clinical signs compatible with *T. equi* infection	Present	Absent
History of tick exposure	Present	Absent/Not documented
IFAT	Positive	Negative
PCR for *T. equi*	Positive	Negative
Blood smear	Evaluated (not used for inclusion)	Negative
Antiparasitic/supportive treatment before sampling	None	None
	Compatible with infection	Within reference intervals

TEQ, horses infected with *T. equi*; CTR, healthy horses.

**Table 5:** Clinical scoring system (CSS) used for the assessment of disease severity.

Variables	Scoring	
	0	1
Poor performance	Absent	Present
Hyperthermia	Absent (37.5–38.5 °C)	Present (>38.5 °C)
Leukocytosis	Absent (5.5–12 × 10^9^/µL)	Present (>12 × 10^9^/µL)
Mucous membranes	Normal	Altered

## Data Availability

The data supporting the findings of this study are not publicly available but may be available from the corresponding author upon reasonable request.
